# The control of endopolygalacturonase expression by the sugarcane RAV transcription factor during aerenchyma formation

**DOI:** 10.1093/jxb/ery362

**Published:** 2019-01-03

**Authors:** Eveline Q P Tavares, Amanda P De Souza, Grayce H Romim, Adriana Grandis, Anna Plasencia, Jonas W Gaiarsa, Jacqueline Grima-Pettenati, Nathalia de Setta, Marie-Anne Van Sluys, Marcos S Buckeridge

**Affiliations:** 1 Departamento de Botânica, Instituto de Biociências, Universidade de São Paulo, SP, Brazil; 2 LRSV, Laboratoire de Recherche en Sciences Végétales, UMR5546, Université Paul Sabatier Toulouse III/CNRS Castanet-Tolosan, France; 3 Tau Bioinformatics, São Paulo, SP, Brazil; 4 Centro de Ciências Naturais e Humanas. Universidade Federal do ABC, São André, SP, Brazil

**Keywords:** Aerenchyma, sugarcane, endopolygalacturonase, ethylene, RAV, *Saccharum*, 2G ethanol

## Abstract

The development of lysigenous aerenchyma starts with cell expansion and degradation of pectin from the middle lamella, leading to cell wall modification, and culminating with cell separation. Here we report that nutritional starvation of sugarcane induced gene expression along sections of the first 5 cm of the root and between treatments. We selected two candidate genes: a RAV transcription factor, from the ethylene response factors superfamily, and an endopolygalacturonase (EPG), a glycosyl hydrolase related to homogalacturonan hydrolysis from the middle lamella. *epg1* and *rav1* transcriptional patterns suggest they are essential genes at the initial steps of pectin degradation during aerenchyma development in sugarcane. Due to the high complexity of the sugarcane genome, *rav1* and *epg1* were sequenced from 17 bacterial artificial chromosome clones containing hom(e)ologous genomic regions, and the sequences were compared with those of *Sorghum bicolor*. We used one hom(e)olog sequence from each gene for transactivation assays in tobacco. *rav1* was shown to bind to the *epg1* promoter, repressing β-glucuronidase activity. RAV repression upon *epg1* transcription is the first reported link between ethylene regulation and pectin hydrolysis during aerenchyma formation. Our findings may help to elucidate cell wall degradation in sugarcane and therefore contribute to second-generation bioethanol production.

## Teaser

Aerenchyma formation in sugarcane roots is controlled by an ethylene-related RAV transcription factor. It binds to the promoter of an endogalacturonase and apparently controls cell separation.

## Introduction

Aerenchyma occurs in parenchymatic tissues of leaves, stems, or roots. It is characterized by enlarged and interconnected intercellular spaces filled with gas (Evans, 2003). It can be a result of schizogenous or lysigenous processes. The former does not involve cell death and is a result of the enlargement of intercellular spaces. The second involves cell separation, but the gas spaces result from programmed cell death (PCD; Bouranis *et al.*, 2007).

Lysigenous aerenchyma can be constitutive or induced by abiotic stresses such as nutritional starvation, osmotic stress, and hypoxia (Fagerstedt, 2010). They have been reported in a broad diversity of plants (Jackson *et al.*, 1985; Drew *et al.*, 2000; Aschi-Smiti *et al.*, 2003; Colmer, 2003; Fan *et al.*, 2003; Shimamura *et al.*, 2010; Yamauchi *et al.*, 2013). In maize, for instance, aerenchyma development requires an environmental inducer (Rajhi *et al.*, 2011), whereas it is constitutive in other plants such as rice (Justin and Armstrong, 1991), sorghum (Promkhambut *et al.*, 2011), sugarcane (Begum *et al.*, 2013; Leite *et al.*, 2017; Tavares *et al.*, 2018), and some maize relatives (Abiko *et al.*, 2012).

The initiation of lysigenous aerenchyma is marked by PCD and cell wall (CW) hydrolysis. While PCD events are well characterized (Gunawardena *et al.*, 2001), there is a gap in understanding the regulation of CW hydrolysis during aerenchyma formation. CW changes require glycosyl hydrolases such as xylanases and cellulases, as well as modifications in xyloglucans and degradation of mixed β-glucans (Rajhi *et al.*, 2011; Leite *et al.*, 2017). However, it is known that the first CW polysaccharide degraded during aerenchyma development is pectin (Gunawardena *et al.*, 2001; Leite *et al.*, 2017). Other CW degradation events (e.g. fruit ripening) also require pectin degradation, suggesting that this step is crucial for triggering the subsequent CW modifications (Tavares *et al.*, 2015).

In maize roots, a decrease in the degree of esterification of homogalacturonan, the most abundant pectin in the middle lamella, occurs early during aerenchyma development. Such an alteration becomes visible in the cortex, being observed even before ultrastructural changes (Gunawardena *et al.*, 2001). The degradation of homogalacturonan in the middle lamella was also observed during aerenchyma formation in sugarcane roots, and it seems to be associated with the process of cell separation (Leite *et al.*, 2017). Also, gene expression and enzymatic activities of pectinases have been reported in several studies (Bragina *et al.*, 2001; Rajhi *et al.*, 2011; Yamauchi *et al.* 2013, Tavares *et al.*, 2018).

It is known that the plant hormone ethylene is the main trigger in both induced and constitutive aerenchyma (Shiono *et al.*, 2008; Yukiyoshi and Karahara, 2014). Several families of transcription factors, including ethylene response factors (ERFs) NAC [for NAM (no apical meristem), ATAF, CUC (cup-shaped cotyledon-apical meristem)], and MYB (first described as an avian MYeloBlastosis virus gene] have been identified as participating in aerenchyma development (Rajhi *et al.*, 2011). Members of these families are thought to regulate gene expression related to lignin and phenylpropanoid biosynthesis (Legay *et al.*, 2010; Nakano *et al.*, 2015; Soler *et al.*, 2016), but their role in aerenchyma formation is still unknown.

The identification of key players in aerenchyma formation may uncover an unexplored biotechnological potential. This is because pectins, which even in relatively lower amounts in grass CWs, are among the subtleties associated with recalcitrance in hydrolysis (De Souza *et al.*, 2015, Latarullo *et al.*, 2016). The acquisition of endogenous control of pectin degradation from the inside out could facilitate the deconstruction of CWs for second-generation (2G)-bioethanol production (Grandis *et al.*, 2014; Tavares *et al.*, 2015). This would be achieved by triggering the CW degradation before harvesting, so that biomass would require fewer pre-treatments and lower amounts of enzymatic cocktails to be degraded (Tavares *et al.*, 2015; Latarullo *et al.*, 2016).

Here, we evaluated whether previously described transcription factors indicated to be related to aerenchyma (Rajhi *et al.*, 2011) could directly regulate key genes during PCD and CW modifications in sugarcane roots. The gene expression profile and some anatomical features along the 5 cm long sugarcane root tip revealed a continuous development of aerenchyma. We found that constitutive aerenchyma formation in sugarcane can be delayed—but not blocked—as a result of nutrient availability. Genes encoding an endopolygalacturonase (EPG) and a RAV transcription factor family are differentially expressed across the root segments and highly accumulated in starved plants. We sequenced the corresponding genomic regions from the sugarcane R570 cultivar and compared it with the corresponding orthologous regions from *Sorghum bicolor*. The analysis of the promoter region showed that the EPG gene displays RAV-binding sites, and transactivation assays indicated that the RAV transcription factor represses *epg1* gene expression. Our results support the hypothesis that the two isolated genomic regions (*rav1* and *epg1* loci) contain essential genes that control early pectin degradation during aerenchyma formation in sugarcane roots. Therefore, these results highlight the biotechnological potential for further sugarcane genetic manipulation towards an improved 2G-ethanol feedstock.

## Materials and methods

### Plant material, experimental treatments, and harvesting

To evaluate whether nutrients can affect the constitutive aerenchyma of sugarcane, we grew plants in two distinct nutrient availability conditions. Culm sections of sugarcane (cv. SP80-3280) 8 cm in length containing one bud each were collected from a field in Piracicaba, São Paulo, Brazil. Culm sections were planted into 15 × 10 cm trays containing vermiculite and maintained in the greenhouse. After 14 d, 10–15 cm tall juvenile plants were transferred to 15 liter pots with drainage holes containing vermiculite mixed with 40 g of NPK (30:20:30) fertilizer.

Plants were subjected to two different nutrient availability levels (full nutrient and nutrient starvation) from October 2011 to January 2012. In the full nutrient availability treatment (+Hoagland), plants received 100 ml of Hoagland solution (9 mM KH_2_PO_4_, 5 mM KCl, 5 mM CaCl_2_·H_2_O, 80 mM NH_4_NO_3_, 2.0 μM MnCl_2_·4H_2_O, 0.23 mM H_3_BO_3_, 200 μM FeSO_4_, 3.6 μM ZnCl_2_, 1.5 μM CuCl_2_, 0.5 μM H_2_MoO_4_·H_2_O, and 0.2 μM Na_2_EDTA) weekly. In nutrient starvation treatment plants were irrigated only with distilled water instead of Hoagland solution. Plants were kept outside the greenhouse and subjected to natural periodic rain. During this period, average temperature and precipitation ranged from 19 °C to 22 °C and from 130 mm to 250 mm, respectively (INPE, National Institute for Space Research; www.cptec.inpe.br).

Tiller roots of 4-month-old plants were harvested and divided into five segments of 1 cm each from the apex of the root. The segments were named S1–S5, from the root tip onwards. This followed the procedure described by Leite *et al.* (2017). For every three plants, all tiller roots were removed, immediately segmented, and frozen in liquid nitrogen, constituting a biological replicate. In total, three biological replicates in each treatment were collected. The samples were kept at –80 °C until the analyses. Five tiller roots of each treatment were fixed in 70% ethanol for determination of aerenchyma area.

### Determination of aerenchyma cortical area

Cross-sections of tiller root segments were used to evaluate the extent of aerenchyma formation, which was defined in this work as the percentage of the aerenchyma cross-sectional area occupying the cortex of the root. The sections were stained with a solution of 1% safranin and 0.1% Astra blue. Sections were examined under an Olympus BX51light microscope with an Aplanat Achromat 1.4 condenser, and photographed using an Olympus Evolt E330 digital camera (Olympus America Inc.^®^). The area occupied by aerenchyma within the cortex was measured using the software ImagePro (MediaCybernetics®, v.6.3, Rockville, MD, USA).

### RNA extraction and reverse transcription–quantitative PCR (RT–qPCR) assays

Root segment samples were ground to a fine powder in liquid nitrogen using a pre-cooled mortar and pestle. A 200 mg aliquot of the pulverized tissue was transferred to a 1.5 ml tube and homogenized in 1.5 ml of Trizol (Invitrogen by Life Technologies^®^). Total RNA extraction followed the manufacturer’s instructions. RNA pellets were suspended in 30 μl of diethylpyrocarbonate-treated water. RNA samples were quantified in a spectrophotometer (NanoDrop; ThermoFisher Scientific^®^), and RNA integrity was evaluated in a 0.8% agarose gel. Total RNA was treated with Amplification Grade DNase I (ThermoFischer Scientific^®^) following the manufacturer’s procedures. An aliquot of treated RNA was used in qPCR to rule out DNA contamination using the GAPDH primer (Iskandar *et al.*, 2004). The cDNA synthesis was performed using the SuperScript First-Strand Synthesis System for RT–PCR (Invitrogen by Thermo Fischer Scientific^®^).

Sixteen pairs of primers were designed to evaluate the transcriptional levels of target genes (Supplementary Table S1 at *JXB* online). All sugarcane-assembled sequences used for primer design were downloaded from the Sugarcane EST database (http://sucest-fun.org/; Vettore *et al.*, 2001).

Primers were designed using Primer 3 (http://frodo.wi.mit.edu/primer3/) and the following parameters: *T*_m_ 58–60 °C, 40–60% GC. For qPCR primers, the amplicon range was from 50 bp to 150 bp. Primer efficiencies were calculated by standard curve dilutions, and only those with efficiency {[10(–1/slope)–1]×100} within the 90–110% range were used. All RT–qPCRs were made in technical triplicates using SYBR Green PCR Master Mix (Thermo Fischer Scientific^®^). PCR amplification was monitored and analyzed with a 7300 Real-Time PCR System (Applied Biosystems by Thermo Fischer Scientific^®^). The cycling amplification consisted of denaturation at 95 °C for 5 min, followed by 40 cycles of denaturation at 95 °C, primer annealing at 60 °C, and extension at 72 °C for 1 min each step.

Three reference genes [polyubiquitin (SCCCST2001G02.g), rRNA 60S (SCJFRZ2009G01.g), and ubiquitin (SCBGLR1002D06.g); Supplementary Table S1] were selected for their unaltered expression across the samples using the GeNormPLUS tool (Biogazelle, Zwijnaarde, Belgium) (Hellemans *et al.*, 2007). mRNA expression was determined using qBasePLUS 2.0 software.

### BAC SHCRBa library screening, sequencing, and assembly

Bacterial artificial chromosomes (BACs) were selected from the SHCRBa sugarcane R570 library through 3D pool screening by qPCR. The same primer set used to amplify *rav1* and *epg1* (designed using SCEPFL3083G08.g and SCAGLR2011B02.g ESTs, respectively) was used for the screening (Supplementary Table S1). The construction and screening of 3D pools were performed as previously described (Adam-Blondon *et al.*, 2005; de Setta *et al.*, 2014). Sequencing and gene annotation of the 17 selected BACs (eight with the *epg1* sequence and nine with the *rav1* sequence) were performed according to de Setta *et al.* (2014) All sequences were submitted to the NCBI database under BioProject ID PRJNA406888.

### Synteny and phylogenetic analyses using SHCRBa BACs, the *S. bicolor* genome sequence, and *Saccharum officinarum*, and *Saccharum spontaneum* amplicons

The search for target gene orthologs was performed using unmasked *S. bicolor* genome v2.1 (Paterson *et al.*, 2009) available on Phytozome v9.1 (http://www.phytozome.net/) (Goodstein *et al.*, 2012) with the e-value cut-off of e^–50^. Sequences from hom(e)ologous genes were analyzed comparatively concerning their start and stop codon positions, as well as their intron number and positions through alignment using MUSCLE software (Edgar, 2004). Phylogenetic analyses were carried out using *S. bicolor* and sugarcane coding sequence alignment with the best substitution model through the Neighbor–Joining method, and 1000 bootstrap replicates on MEGA v.5.2.2 (Tamura *et al.*, 2011).

Similarity analysis between *S. bicolor* and sugarcane genes was performed on sequences ranging up to 100 kb upstream and downstream of target genes. The prediction of sugarcane genes in the BAC sequences followed two criteria: (i) query sugarcane sequences were aligned through BLASTx, and only those presenting matches with an e-value cut-off from e-10 to e-15 were kept; and (ii) predicted sequences with no annotation, with no known protein domains, and present in only one BAC from each set were excluded. Collinear genes were defined as those with minimum coverage and identity of 90% and 75%, respectively. Orthologs between sugarcane and *S. bicolor* were identified through loci gene conservation within the analyzed fragment.

Transposable element (TE) mapping was performed only for sugarcane sequences and identified using the RepeatMasker search engine and Viridiplantae databank. The hits presenting a minimum score of 250 and 3 kb length were represented on the synteny analysis. Further classification was performed with an in-house TE data set as described in Domingues *et al.* (2012).

Concatenated coding sequences from conserved blocks around the target genes were aligned, and phylogenetic reconstruction using Neighbor–Joining was inferred from the Tajima–Nei method and 1000 bootstrap replications.

### *Cis*-element prediction, plasmid constructions, and *Agrobacterium tumefaciens* transformation

The *rpg1* promoter sequence (2 kb upstream of the predicted start codon) was loaded onto the PLACE tool (database of Plant Cis-acting Regulatory DNA Elements) (Prestridge, 1991) for *in silico* mapping of sequences potentially recognized by RAV transcription factors.

A 1.2 kb segment of *rav1* (gene) and *epg1* (promoter, EPG1pm) were PCR amplified using primers based on the SHCRBa_021A10 and SHCRBa_224P16 BAC sequences, respectively (Supplementary Table S1). The reaction mix was set up with 0.2 mM dNTPs (Promega), 2.5 mM MgCl_2_, 3% DMSO, 0.2 µM of each primer, and 1 U of Phusion High-Fidelity DNA Polymerase (ThermoFischer Scientific^®^) in 1× GC buffer. The amplification conditions were denaturation at 98 °C for 35 s, followed by 30 cycles of denaturation at 98 °C, annealing for 30 s, and extension for 40 s at 72 °C. The annealing temperature followed a touchdown procedure starting at 61.5 °C and decreasing 0.3 °C at each cycle. The reaction was finished with the last step of extension at 72 °C for 10 min. Amplicons were ligated into a pENTR™/D-TOPO vector (Life Technologies by ThermoFischer Scientific^®^) and used to transform chemically competent *Escherichia coli* DH5α. Plasmids were extracted using the GeneJET Plasmid Miniprep Kit (ThermoFischer Scientific^®^) and sequenced. Sequencing reactions were undertaken with 150 ng of template, 0.5 μM of primer, and 3700 big dye terminator mix version 3.1, and read in an ABI 3730 DNA sequencer (Life Technologies by ThermoFischer Scientific^®^). Activator and reporter constructs harboring fragments corresponding to the genomic sequence of *rav1* and EPG1pm, respectively, were cloned into pFAST-G02 (Shimada *et al.*, 2010) and pKGWFS7 (Karimi *et al.*, 2002) using Gateway^®^ LR Clonase^®^ II (Life Technologies by ThermoFischer Scientific^®^). Final constructs were used to transform electrocompetent *Agrobacterium tumefaciens* GV3101 cells (Wise *et al.*, 2006).

### Infiltration of *Nicotiana benthamiana* leaves

One single colony of each of the transformed *A. tumefaciens* systems (harboring activator and reporter constructs) was used to inoculate 2 ml of LB medium supplemented with gentamycin (20 µg ml^–1^) to select the bacterial strain and spectinomycin (100 µg ml^–1^) to select the plasmid. The pre-culture was incubated at 28 °C at 250 rpm for 20–24 h. *Nicotiana benthamiana* plants were grown for 4–5 weeks at 22–25 °C, with a 16 h/8 h photoperiod (12 µmol m^–2^ s^–1^ of light intensity) and 80% humidity. After *A. tumefaciens* infiltration, the plants were kept in this same condition for 2 d. Reporter activity was followed by β-glucuronidase (GUS) assay performed with 10 mM 4-methylumbelliferyl β-d-galactopyranoside. The enzymatic reaction was performed at 37 °C for 2 h 30 min, with measurements every 5 min in a microplate reader (Tristar LB 941 from Berthold Technologies) using the Mikrowin 2000 software. Culture preparation, infiltration, and reporter activity of *A. tumefaciens* were as described in Soler *et al.* (2016). Three independent experiments with three biological replicate each were performed. Protein concentration was quantified by the Bradford method (Bio-Rad) using BSA as a standard. GUS activities were normalized by the total protein amount.

### Statistical analysis

Statistically significant differences were evaluated using two-way ANOVA followed by Tukey test (*P* ≤ 0.1), defining root segments, treatment, and the interaction between segment and treatment as fixed factors (*n*=3). GUS activity from tobacco protein extracts was analyzed using Student’s *t*-test (*P*<0.05). All analyses were performed with the software GraphPad^®^, version 6.

## Results

### Nutrient starvation increases aerenchyma formation and changes in the transcriptional profile

**Fig. 1.**
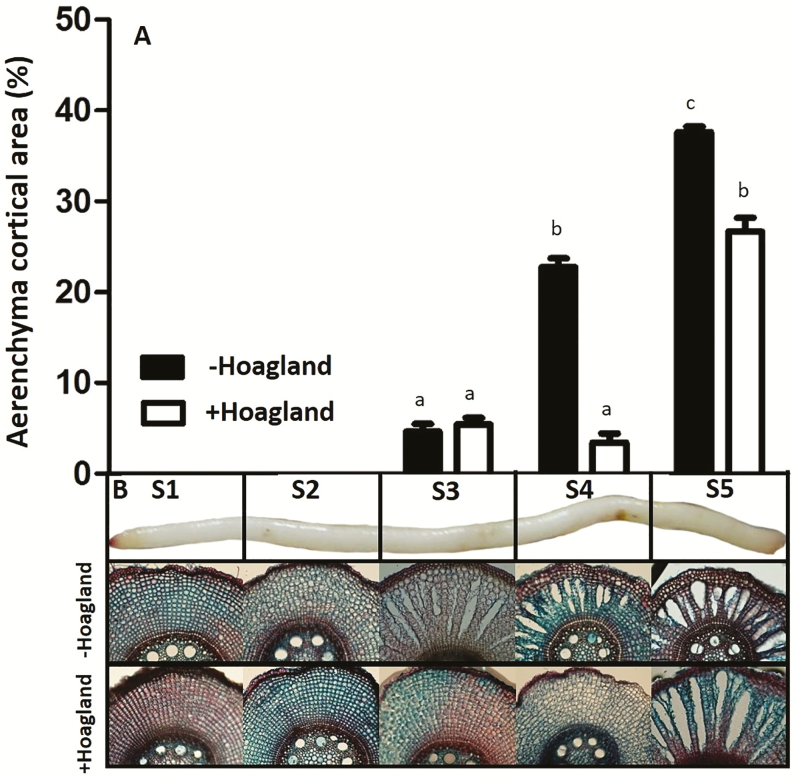
Aerenchyma formation upon different nutrient availability. (A) Cortex cross-sectional area occupied by the aerenchyma upon high (+Hoagland) or low (–Hoagland) nutrient availability. (B) Sugarcane root segments and cross-sections. Measurements were performed in five different roots in three pools of five plants each. Different letters denote statistically significant differences (*P*<0.1) between root segments and treatments.

In both nutrient treatments, aerenchyma formation was not visible up to 3 cm from the root tip (Fig. 1). From this point on, the opening of gas spaces increased from 5% (on segment 3, S3) to 23% (on S4) in the –Hoagland plants. At S5, the aerenchyma filled ~38% of the cortical cross-sectional area of the sugarcane root in this same treatment. In plants irrigated with nutrient solution (+Hoagland), the aerenchyma area was 6.7- and 1.4-fold smaller, respectively, than the corresponding S4 and S5 from plants grown under starvation conditions (Fig. 1).

**Fig. 2.**
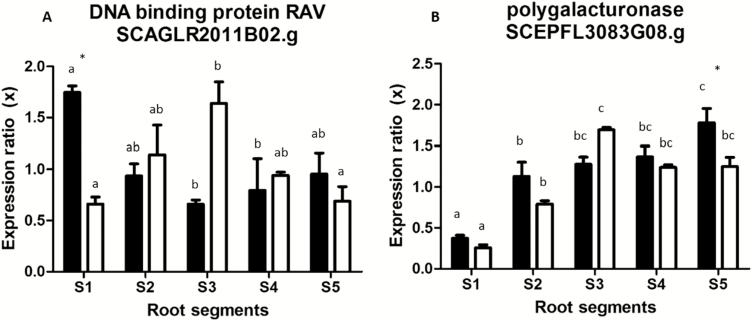
RT–qPCR of transcripts corresponding to transcription factor RAV (A) and endopolygalacturonase (EPG) (B). The *y*-axis is the relative expression ratio ±SE between –Hoagland (black bars) and +Hoagland samples (plants grown with available nutrients; white bars). Reactions were done in triplicate and using three biological replicates as a result of pools of roots from three plants each. Different letters denote statistically significant differences between root segments and treatments, and asterisks show statistically significant differences in the same segment from different treatments (*P*<0.1).

Targets for RT–qPCR were selected among transcripts related to CW degradation, PCD, and ethylene signaling that have been expressed during sugarcane root development (Lima *et al.*, 2001) and maize root aerenchyma (Rajhi *et al.*, 2011). No significant alterations of the levels of MYB (SCRLLR1059G12.g) and NAC genes (SCCCLR2003C07.g) were observed along the root sections or between nutrient conditions (Supplementary Fig. S1). Conversely, two genes coding for ERFs [ethylene response factor 1 (ERF1) and RAV] showed differentially expressed patterns during aerenchyma formation. ERF1 (SCCCCL4002B07.g) was mainly expressed in the root apex (root segment 1) in both nutrient treatments, decreasing its mRNA levels in S2 and maintaining similar levels towards S5 (Supplementary Fig. S1). The accumulation of *rav1* mRNA (SCAGLR2011B02.g) was almost 3-fold higher on S1 of –Hoagland and peaked at S3 of +Hoagland plants (Fig. 2A).

The mRNA levels of the PCD-related genes calcineurin (SCCCLB1004G05.g) and calmodulin (SCCCLR2003G10.g) were lower in the root apex, increasing significantly on the subsequent root segments on +Hoagland treatment. mRNA levels of cysteine protease (SCCCLB1023F09.g) were constant throughout the root segments, peaking only on S5 of – Hoagland plants (Supplementary Fig. S1).

The analysis of CW-related genes revealed that the mRNA corresponding to one expansin (SCVPRT2075H10.g) peaked on the root apex, reaching higher levels on –Hoagland plants, whereas xyloglucan transglycosylase/hydrolase expression remained stable along the root (SCBGLR1002B06.g) (Supplementary Fig. S2).

The expression of the gene coding for an EPG (SCEPFL3083G08.g) increased from S1 to S3 in both treatments, reaching higher levels on S5 on –Hoagland plants (Fig. 2B). Pectinacetylesterase mRNA (SCAGRT2038D08.g) reached higher levels from S2 on and remained stable towards S5 (Supplementary Fig. S2). Transcripts corresponding to an α-arabinofuranosidase (SCQSRT1034D03.g) and an exoglucanase (SCEQRT1029E06.g) showed higher levels in S1 in –Hoagland plants, decreasing their expression in S2 with a subsequent linear increase towards S5. β-Glucosidase (SCEQHR1082B01.g) mRNA levels were higher on S1 and S2 regardless of nutrient availability (Supplementary Fig. S2).

RAV is an ERF (Nakano *et al.*, 2006), and ethylene signaling and pectin degradation are both hallmarks of aerenchyma formation (Gunawardena *et al.*, 2001; Leite *et al.*, 2017). Although the influence of the other genes analyzed cannot be disregarded, the expression pattern of *rav1* and *epg1* genes coincides with the expected signaling and with the aerenchyma development observed in sugarcane under different nutrient availabilities. Therefore, these two genes were selected for further sequencing and characterization, as described below.

### Analysis of structural features between hom(e)olog sugarcane BACs and the *S. bicolor* ortholog loci

**Fig. 3.**
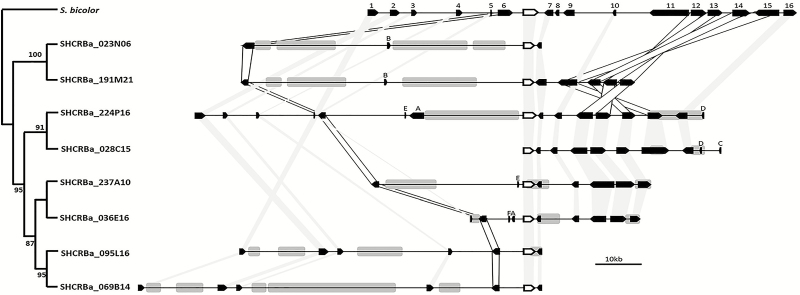
Physical and phylogenetic relationships within ScEPG1 loci from sugarcane BAC sequences compared with the orthologous locus from *S. bicolor* (Chr1: 71.679.279–71.777.845). ScEPG1 hom(e)ologous sequences are represented by white arrows, and the remaining genes are represented by black arrows. Shaded light gray areas indicate collinearity between the target gene and neighboring genes between *S. bicolor* and sugarcane. Shaded white areas indicate syntenic genes between both species, but showing opposite orientation. Transposable elements harboring long terminal repeats (LTRs) are represented by gray boxes. Only those elements >3 kb are shown. Phylogenetic reconstruction using Neighbor–Joining was inferred from the Tajima–Nei method and 1000 bootstrap replications. Numbers and gene annotation, according to *S. bicolor* (1) Sobic.001G525600, similar to fringe protein, putative, expressed; (2) Sobic.001G525500, similar to protein kinase domain-containing protein, expressed; (3) Sobic.001G525400; similar to protein kinase, putative, expressed; (4) Sobic.001G525300, weakly similar to expressed protein; (5) Sobic.001G525200, hypothetical gene; (6) Sobic.001G525100, hypothetical gene; (7) Sobic.001G524900, similar to 50S ribosomal protein L5, chloroplast precursor; (8) Sobic.001G524800, hypothetical gene; (9) Sobic.001G524700, hypothetical gene; (10) Sobic.001G524600, hypothetical gene; (11) Sobic.001G524500, similar to HAD-superfamily hydrolase, subfamily IA, variant 3-containing protein, expressed; (12) Sobic.001G524400, similar to D111/G-patch domain-containing protein, putative, expressed; (13) Sobic.001G524300, similar to expressed protein (diacylglycerol kinase catalytic domain); (14) Sobic.001G524200, similar to protein kinase domain-containing protein, expressed; (15) Sobic.001G524100, similar to expressed protein; (16) Sobic.001G524000; hypothetical gene; (target gene) Sobic.001G525000, similar to polygalacturonase, putative, expressed. Letters indicate gene sequences that were annotated in sugarcane and had no orthologous sequence in *S. bicolor*: (A) Hypothetical gene; (B) transcription elongation factor (TFIIS) family protein; (C) oxysterol-binding_family_protein; (D) pectate lyase.

**Fig. 4.**
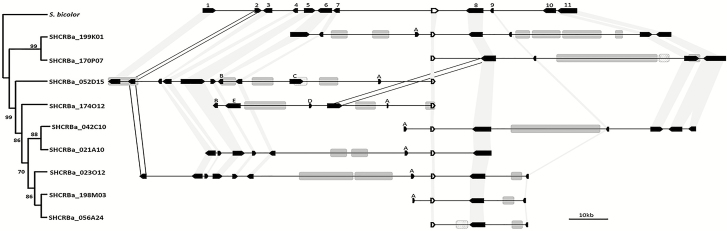
Physical and phylogenetic relationships within *rav1* loci from sugarcane BAC sequences compared with the orthologous locus from *S. bicolor* (Chr3: 60.193.612–60.305.674). *rav1* hom(e)ologous sequences are represented by white arrows, and the remaining genes are represented by black arrows. Shaded light gray areas indicate collinearity between the target gene and neighboring genes between *S. bicolor* and sugarcane. Shaded white areas indicate syntenic genes between both species, but showing opposite orientation. Transposable elements harboring long terminal repeats (LTRs) and DNA transposable elements are represented by gray and dotted boxes, respectively. Only those elements >3 kb are shown. Phylogenetic reconstruction using Neighbor–Joining was inferred from the Tajima–Nei method and 1000 bootstrap replications. Numbers and gene annotation, according to *S. bicolor* (1) Sobic.003G265900, serine/threonine protein kinase; (2) Sobic.003G265800, similar to Os12g0102600 protein; (3) Sobic.003G265700, serine/threonine protein kinase; (4) Sobic.003G265600, non-specific serine/threonine protein kinase; (5) Sobic.003G265500, peptidyl-tRNA hydrolase; (6) Sobic.003G265400, similar to Os01g0693800 protein; (7) Sobic.003G265300, similar to putative lectin-like receptor kinase; (8) Sobic.003G265100, hypothetical gene; (9) Sobic.003G265000, weakly similar to putative uncharacterized protein P0034C09.35; (10) Sobic.003G264900, protein RHG1a-like; (11) Sobic.003G264800 similar to Os01g0692600 protein; (target gene) Sobic.003G265200, transcription factor RAV-like. Letters indicate gene sequences that were annotated in sugarcane and had no orthologous sequence in *S. bicolor*: (A) UDP glucosyl transferase; (B) hypothetical gene; (C) fascilin; (D) ATPase.

We identified and selected eight BACs for *epg1* and nine for *rav1* (Figs 3, 4). All sequenced BACs had the full-length candidate gene sequence, and each primer set identified only a single locus. The eight *epg1* and nine *rav1* coding sequences were at least 99% and 96% identical to each other’s hom(e)olog, respectively.

A high collinearity was observed between sugarcane BAC sequences, suggesting that they are hom(e)ologous regions. Collinearity was higher for *rav1* BACs, in which the sugarcane ortholog genes were identified for all of the 11 genes within the *S. bicolor* locus despite some rearrangements between sugarcane and *S. bicolor* genes (light gray shaded areas; Figs 3 and 4). Conversely, a sugarcane ortholog for two of the hypothetical genes from *S. bicolor* (genes 8 and 9; Fig. 3), within the EPG gene locus, were missing. A four gene block (12–15, ~26 kb) displayed an inverted orientation to *S. bicolor*. Another sugarcane hom(e)olog inversion was found in all sugarcane BACs (hypothetical gene, numbered as 6; Fig. 3).

Phylogenetic trees for *epg1* and *rav1* displayed an early diverged and well-supported clade harboring two BACs. These are SHCRBa_023N06 and SHCRBa_191M21 for BACs with *epg1* (Fig. 3) and SHCRBA_170P07 and SHCRBa_199K01 for BACs with *rav1* (Fig. 4). To evaluate whether this grouping pattern could reflect the unequal chromosomal contribution of the parental *S. officinarum* and *S. spontaneum* genomes to modern sugarcane (D’Hont, 2005), we evaluated the nucleotide diversity of *S. officinarum*, *S. spontaneum*, and modern sugarcane *rav1* and *epg1* partial sequences. This could indicate which of the parent species is the provider of some of the *rav1* and/or *epg1* hom(e)olog sequences.

All the *rav1* sequences from BACs grouped with *S. officinarum* sequences (Supplementary Fig. S3), including the most divergent *rav1* genes, from SHCRBa_199K01 and 170P07 BACs. On the other hand, *epg1* BACs grouped with both *S. officinarum* and *S. spontaneum*. Interestingly, a specific and well-supported *S. spontaneum* clade grouped with the most divergent *epg1* BACs SHCRBa_023N06 and 191M21 (Supplementary Fig. S4).

### Transcriptional and structural features from different *rav1* and *epg1* hom(e)ologs

**Fig. 5.**
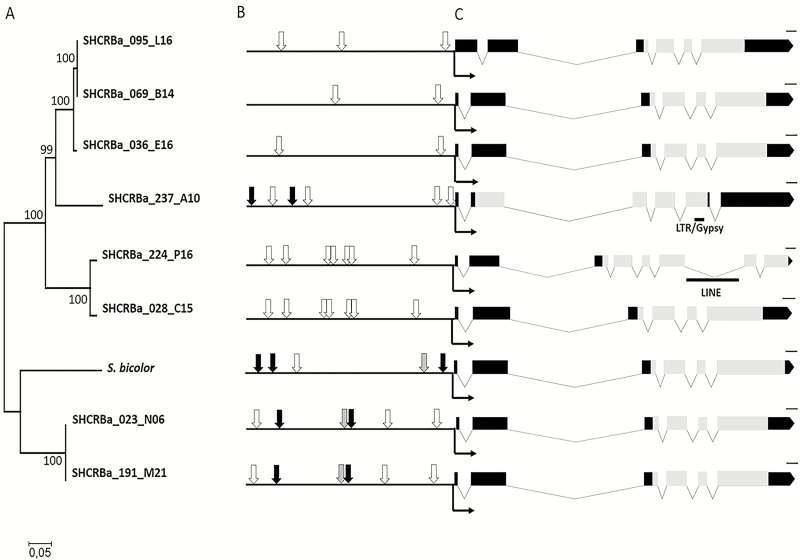
Schematic representation of ScEPG1 gene structure and putative promoter from hom(e)ologous sequences. (A) Phylogenetic reconstruction upon multiple alignments using the 2 kb upstream translation start site (black L-shaped arrow in C) by Neighbor–Joining inferred from the Tajima–Nei method using 1000 bootstrap replicates. (B) Putative RAV- and AP2/ERF-binding sites (white and black arrows, respectively). Gray arrows point to both binding sites in tandem. (C) ScEPG1 gene structure. Black squares and lines indicate exons and introns, respectively. Gray squares show the GH28 domain from the predicted protein, and black lines represent transposable elements within the sequences. To construct this representation, the tool Exon-Intron Graphic Maker was used. Scale bars correspond to 100 bp.

The RAV transcription factor showed AP2 and B3 DNA-binding domains (Fig. 5), each of them responsible for binding to two specific *cis*-element promoters (Kagaya *et al.*, 1999). A 2 kb sequence upstream of the translation start site from the eight *epg1* genes and a *S. bicolor* ortholog had RAV AP2-binding sites in all promoters analyzed (Fig. 5B). Both *cis*-elements were found in tandem in *S. bicolor* and also within the two most divergent BACs (Fig. 5B), which seems not to be a functional conformation for RAV binding (Kagaya *et al.*, 1999). No B3-binding sites were identified. Sites for ERF binding occurred in three out of eight *epg1* BAC sequences and in a *S. bicolor* ortholog (black arrows; Fig. 5B).

The *epg1* promoter harboring the highest number of putative AP2-binding sites (from the SHCRBa_028C15 BAC sequence) was selected as the candidate for transactivation assays to evaluate whether RAV1 could interact with the pectinase gene promoter *in vivo*.

**Fig. 6.**
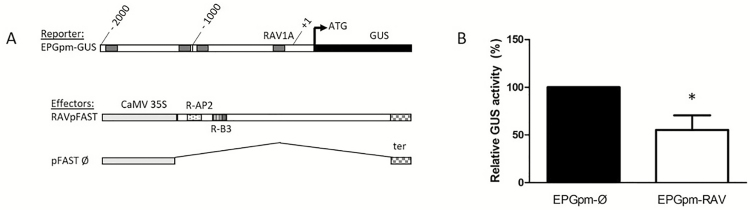
Effects of RAV1 (RAVpFAST) on transcriptional activation of the ScEPG1 promoter (EPGpm-GUS) in vivo. (A) Schematic maps of the reporter and effector constructs. CaMV35S, *Cauliflower mosaic virus* 35S promoter; GUS, uidA coding region; +1, transcription start site; RAV1A, putative sites for AP2 binding (gray boxes); ter, nopaline synthase terminator (box with grids); R-AP2, AP2 DNA-binding domain of ScRAV1 (dotted box); R-B3, B3 DNA-binding domain of ScRAV1 (box with vertical lines). (B) Results from co-transfection experiments in leaves of *Nicotiana benthamiana*. *Agrobacterium tumefaciens* containing effector and reporter constructs were co-infiltrated into *N. benthamiana* leaves. Data represent mean values and SDs of three independent experiments, each containing three biological replicates. GUS activity is expressed as a percentage relative to control plants (pFAST Ø, ‘empty’ vector co-transfected with reporter construct EPGpm). *The change in GUS activity due to RAV1 is statistically significant relative to control (Student’s *P*<0.05).

As suggested by the presence of putative binding sites for RAV1 on the *epg1* promoter sequence (Fig. 5B), the co-transfection of both reporter and effector constructs harboring the *rav1* coding sequence into *N. bethamiana* leaves led to a statistically significant change in the GUS expression level compared with the empty vector control (Fig. 6). In three independent experiments, the infiltration with the *rav1* sequence resulted, on average, in a 50% decrease in the activation of the *epg1* promoter.

## Discussion

### Cell wall changes and gene expression regulation during aerenchyma formation

Duri ng the formation of lysigenous aerenchyma, the first step of CW modifications relies on cell separation and expansion, which are likely to occur as a result of pectinase (Gunawardena *et al.*, 2001; Leite *et al.*, 2017) and expansin actions, respectively (Tavares *et al.*, 2015, 2018). Such modifications in CWs are also known to be triggered by ethylene (Shiono *et al.*, 2008; Yukiyoshi and Karahara, 2014, Tavares *et al.*, 2018). Coincident with such alterations, our results showed high expansin mRNA levels prior to aerenchyma formation, followed by a progressive increase in EPG mRNA expression along the root segments. Also, higher expression of two ERF mRNAs on S1 (Figs 2, 3A; Supplementary Figs S1, S2) was observed. This expression pattern was observed mainly in plants under nutrient starvation, where the aerenchyma development was increased (Figs 1, 2A).

We also found high expression of exoglucanase and arabinofuranosidase genes on S1 in both nutrient treatments (Supplementary Fig. S2). However, the expression of these genes does not coincide with aerenchyma development (Fig. 1). Although the detection of mRNA related to these genes during aerenchyma formation has also been previously described (Rajhi *et al.*, 2011; Tavares *et al.*, 2018), the characterization of CWs during aerenchyma development in sugarcane roots does not support cellulose or arabinoxylan degradation (Leite *et al.*, 2017). Furthermore, arabinoxylan and cellulose are thought to be associated with the pectin matrix within the wall (Buckeridge *et al.*, 2017), so that it is unlikely that cellulase and arabinofuranosidase activities would occur before the pectinolytic attack.

The expression pattern of expansin and EPG (Fig. 2; Supplementary Fig. S2) corroborates both the sequence of events that was proposed for sugarcane CW degradation (De Souza *et al.*, 2013) and the degradation pattern of homogalacturonan in the middle lamella (Gunawardena *et al.*, 2001; Leite *et al.*, 2017). The loosening of the cellulose–xyloglucan domain appears to be concurrent with the attack of pectinases (here represented by EPG) on specific regions of the middle lamella. This process seems to prepare particular areas of the cortex tissue for the formation of CW composites (Supplementary Fig. S1; Leite *et al.*, 2017).

It has been suggested that N or P starvation could lead to increased ethylene sensitivity by up-regulating the signal transduction cascade for ethylene action (He *et al.*, 1994). Here we found that two ERF mRNAs (DNA-binding protein RAV SCAGLR2011B02.g, Fig. 3A, and ERF-like protein 1 SCCCCL4002B07.g, Supplementary Fig. S1) were highly expressed in apical root segments from nutrient-starved plants of sugarcane. Similar observations were made by Rajhi *et al.* (2011) and Tavares *et al.* (2018) for aerenchyma formation in maize and sugarcane roots, respectively. This raised the question of whether these transcription factors could play a role as intermediates in the signaling cascade for the formation of aerenchyma in sugarcane.

The increase in mRNA EPG expression in plants under nutrient starvation followed the peak of mRNA expression of the transcriptional factor RAV (Fig. 2). Other members of the ERF family such as MYB and NAC have been described as possible regulators of CW components such as lignin and phenylpropanoid biosynthesis (Legay *et al.*, 2010; Nakano *et al.*, 2015; Ferreira *et al.*, 2016; Soler *et al.*, 2016). Also, the gene DREB (dehydration-responsive element-binding protein), another ERF family-related gene, was found to regulate promoters of several CW genes during fruit softening, including pectin-related genes (Fan *et al.*, 2016).

The expression patterns of *epg1* and *rav1* observed in our experiment suggest that this transcription factor may play a role in the signaling or regulation of the initial events of CW degradation during aerenchyma development in sugarcane roots. This led us to the decision to characterize and analyze these two genes.

### Characterization of genes related to aerenchyma formation in sugarcane: *epg1* and *rav1*

Following previous observations for other genes in sugarcane (Okura *et al.*, 2016; de Setta *et al.*, 2014), the genomic regions containing *epg1* and *rav1* hom(e)ologous loci were longer than orthologous loci within the *S. bicolor* genome (Figs 3, 4). BAC SHCRBa_224_P16 harbors 12 of 16 orthologous genes observed within *S. bicolor epg1* loci. The sugarcane sequence between genes 2 and 16 is longer than 114 Mb, whereas the *S. bicolor* ortholog fragment is ~95 Mb. As for RAV-harboring BACs, the SHCRBa_023O12 fragment between genes 2 and 9 is >40 kb longer than the *S. bicolor* syntenic region. The intergenic regions were mostly responsible for the overall longer sequences in sugarcane. This was primarily due to long TE sequences, which are probably associated with chromosome rearrangements (Zhang *et al.*, 2011; de Setta *et al.*, 2014), gene sequence gain and losses (Cormack and Peterson, 1994), and other epigenetic phenomena (Slotkin and Martienssen, 2007; Domingues *et al.*, 2012).

Due to the unequal parental genome contribution to modern sugarcane (D’Hont 2005), we hypothesized that the repetitive pattern of the early divergent clade observed in the phylogenetic trees using *rav1* and *epg1* coding sequences (Figs 3, 4, 5A) could represent genomic sequences inherited from one of the modern sugarcane parental species. For both *rav1* and *epg1*, we identified two gene copies that grouped in a more basal clade (Figs 3, 4). Those *epg1* genes were closer to *S. spontaneum* sequences, but *rav1* was more related to those of *S. officinarum* (Supplementary Figs S3, S4). These results might guide further research on the relationship between allele contribution and CW species-specific features related to parental inheritance in the hybrid sugarcane (Ferreira *et al.*, 2016).

### RAV as a possible link between ethylene signaling and pectin degradation

RAV transcription factors possess two DNA-binding domains (AP2 and B3) that can specifically bind to bipartite recognition sequences composed of two unrelated motifs. Our results showed that *epg1* promoters had an RAV1AAT element for RAV AP2 DNA binding, but no B3 *cis*-element was identified in any of the hom(e)ologs (Fig. 5). The transactivation assays showed an interaction between *epg1* and RAV1 (Fig. 8B). The control of expression of a transcription factor on other promoters possessing only one binding site has already been demonstrated (Li *et al.*, 2011) with a relatively lower binding constant (Yamasaki *et al.*, 2004). Thus, the presence of RAV1AAT sites for AP2 binding within the *epg1* promoter supports the interaction of *epg1* with RAV1, even if the B3-binding sites were absent.

The effect of 50% repression of RAV1 on the *epg1* promoter (Fig. 6B) suggests that RAV1 may act as a negative regulator of pectin degradation during the first steps of aerenchyma development. It is known that other members of the ERF family are capable of binding to and repressing several CW-modifying genes, including polygalacturonase, and other pectin-related genes (Fan *et al.*, 2016). The observed decreasing levels of *rav1* mRNA along the segments (Fig. 2A) apparently represent an important regulation feature in aerenchyma formation, since it leads to de-repression of *epg1* transcriptional activity. To our knowledge, this is the first report about a transcription factor linking ethylene signaling and EPG transcriptional activity triggering aerenchyma formation and further CW modifications.

### Relevance for bioenergy

Sugarcane is a major bioenergy crop, being the source of bioethanol that forms a large portion of the liquid fuel market in Brazil (Buckeridge and De Souza, 2017). However, the potential for ethanol production from sugarcane is much higher (Jaiswal *et al.*, 2017). Approximately two-thirds of the available energy is associated with the CWs within the body of the plants and is not yet used (De Souza *et al.*, 2014).

The primary barrier to using CWs for bioethanol production is the relatively high costs of pre-treatment and hydrolysis, and this is directly associated with CW recalcitrance (Soccol *et al.*, 2010, Jordan *et al.*, 2012; De Souza *et al.*, 2014). Although roots are not currently used for ethanol production, understanding the endogenous CW modification steps (such as those taking place during root aerenchyma formation) can help scientists to better cope with CW deconstruction. Finding ways to activate CW modifications through the genetic engineering of CW hydrolysis *in planta* could be key to decrease costs and make 2G technologies commercially viable for bioethanol production on a large scale (Lopez-Casado *et al.*, 2008; Tavares *et al.*, 2015; Ferreira *et al.*, 2016). Remarkably, pectin hydrolysis (e.g. by using EPGs) might lead to significant changes within the wall to increase saccharification efficiency as proposed by Latarullo *et al.* (2016).

Here we show the interaction and regulation of an EPG and RAV1 transcription factor during early events of CW degradation. These results open up possibilities to gain control of these events and use them to facilitate CW hydrolysis in leaves and stem for bioethanol production.

### Conclusions

The expression of *epg1* and *rav1* coincides with aerenchyma formation in sugarcane roots. The full characterization of these two genes showed that the the *epg1* sequence has binding sites for the transcription factor RAV1, suggesting a possible interaction between them *in vivo*. The evaluation of this interaction using transactivation assays demonstrated that RAV1 binds to the *epg1* promoter, repressing its expression. These results might represent an important link between ethylene signaling and pectin degradation that has not yet been described. Additionally, they shed light on what may be the first step to acquiring control of CW hydrolysis in sugarcane through biotechnology, facilitating 2G-bioethanol production.

## Supplementary data

Supplementary data are available at *JXB* online.

**Fig. S1.** RT–qPCR of transcripts corresponding to transcription factors, calcium-related proteins, and cysteine protease.

**Fig. S2.** RT–qPCR cell wall-related transcripts.

**Fig. S3.** Phylogenetic analysis of the 437 bp amplicon obtained from the rav1 gene from sugarcane, *S. officinarum*, and *S. spontaneum*.

**Fig. S4.** Phylogenetic analysis of the 427 bp amplicon obtained from the epg1 gene from sugarcane, *S. officinarum*, and *S. spontaneum*.

## Supplementary Material

Supplementary Table S1

Supplementary Figure S1

Supplementary Figure S2

Supplementary Figure S3

Supplementary Figure S4
