# Supplementary material for: The control of endopolygalacturonase expression by the sugarcane RAV transcription factor during aerenchyma formation
Source: J Exp Bot. 2019 Jan 3;70(2):497–506. doi: 10.1093/jxb/ery362 (PMC6322575; doi:10.1093/jxb/ery362)
Supplement: Supplementary Table S1 [file ery362_suppl_supplementary_table_s1.docx]

**Table S1.** Primers designed from sugarcane assembled sequences (SAS) and annotation

| SAS | Sequence | Annotation |
| --- | --- | --- |
| SCVPLB1020G05.g | TGGCAACACTACGGCACTCC  GCTGCGAGCGAACCAGAGTAA | putative α-L-arabinofuranosidase family |
| SCEQHR1082B01.g | CCTCCAGCGGAATCGTCTTG  GAGTTCGTGCCCATCTTCTTCG | β-glucosidase isozyme 2 precursor |
| SCCCLB1004G05.g | TCGCAGCAGGAGATCGTGTC; TGAGGGAGAACTCGGGGATG | calcineurin subunit B |
| SCCCLR2003G10.g | CCTTCCCAGCCAAGCATCAC; GCGGTCGCGGTAGATTAGATAGATG | calmodulin-like protein |
| SCCCLB1023F09.g | TCCCGCCCCACTTAGATTCC; CCCCTTTCGCTGGTCTCCTC | cathepsin B-like cysteine proteinase 3 |
| SCCCCL4002B07.g | GGCTGTGCAACCCTTCCAAA; GTGGTGGGATGGACGAGGATT | ethylene-responsive factor-like protein 1 |
| SCAGLR2011B02.g | TGGCGCTGTCCATTCCAGT; CATCTCCCGCAACAGAAGCATAC | DNA binding protein RAV |
| SCRLLR1059G12.g | GTTGTGACGGGCGGTTCTTG; GCGGTGTTGAGATGGGAGGA | [MYB DNA-binding domain superfamily](http://blast.ncbi.nlm.nih.gov/Blast.cgi#alnHdr_212722308) |
| SCCCLR2003C07.g | GACAGCTTCCAGACGCACGA; GTGCCAATGCCGTTGTTCCT | [NAC23 NAC type transcription factor, partial](http://blast.ncbi.nlm.nih.gov/Blast.cgi#alnHdr_407232748) |
| SCEQRT1029E06.g | CCTGCTAGCGTTGGGGAGAG; AGTCACCATGGAGGCCCAAG | [exoglucanase precursor](http://blast.ncbi.nlm.nih.gov/Blast.cgi#alnHdr_414872791) |
| SCVPRT2075H10.g | CCGGGGAGGCAGTACTTGAG; GATCTGGGGAAGCACGGCTA | [α-expansin 13 precursor](http://blast.ncbi.nlm.nih.gov/Blast.cgi#alnHdr_212722040) |
| SCAGRT2038D08.g | GCACCAAGCTCCTCCCTGAA; GCCGGGTTACCACTTCCAGA | [putative pectinacetylesterase precursor](http://blast.ncbi.nlm.nih.gov/Blast.cgi#alnHdr_115441565) |
| SCEPFL3083G08.g | GTTGCCGGTCATCCAGAACA; GCGGCTTTGTCAGGGACATT | [polygalacturonase, putative, expressed](http://blast.ncbi.nlm.nih.gov/Blast.cgi#alnHdr_115450425) |
| SCACSB1036D01.g | TGGGCAACAAGGAGATGCAG; ACGCCGTCGATGTACCACAC | [xyloglucan endotransglucosylase/hydrolase 26](http://blast.ncbi.nlm.nih.gov/Blast.cgi#alnHdr_226504064) |
| SCJFRZ2009G01.g | GCGAGTGCCTCACCTTTGAC; TCTTAGGTCCCCTCAGCAGAAC | 60S ribosomal protein L18-2-like |
| SCBGLR1002D06.g | CAGGTCCTGCTGGTGAGGAT  CACCTCCAGCATATGGACTATCAG | ubiquitin-conjugating enzyme E2-17 kDa-like |
| SCCCRZ1002H03.g | CCTCAGAGCCAATCCTTTTCC  ATAACCGCATCAGGTCTCCAAG | putative tubulin alpha-3 chain |
| SCCCLR1072A03.g | CCCTGTGCTGCTCACTGAAG  GTCTCGAACATAATCTGGGTCATCT | ATP synthase subunit O, mitochondrial |
| SCCCLR1069D05.g | CCAGTTCCATTGTCACAAACAAG  TCTCCGGAATCCGTAGCAAA | actin-7-like |
| SCCCST2001G02.g | CCGGTCCTTTAAACCAACTCAGT; CCCTCTGGTGTACCTCCATTTG | polyubiquitin containing 7 ubiquitin monomers |
