# Supplementary figures and images for: The control of endopolygalacturonase expression by the sugarcane RAV transcription factor during aerenchyma formation
Source: J Exp Bot. 2019 Jan 3;70(2):497–506. doi: 10.1093/jxb/ery362 (PMC6322575; doi:10.1093/jxb/ery362)

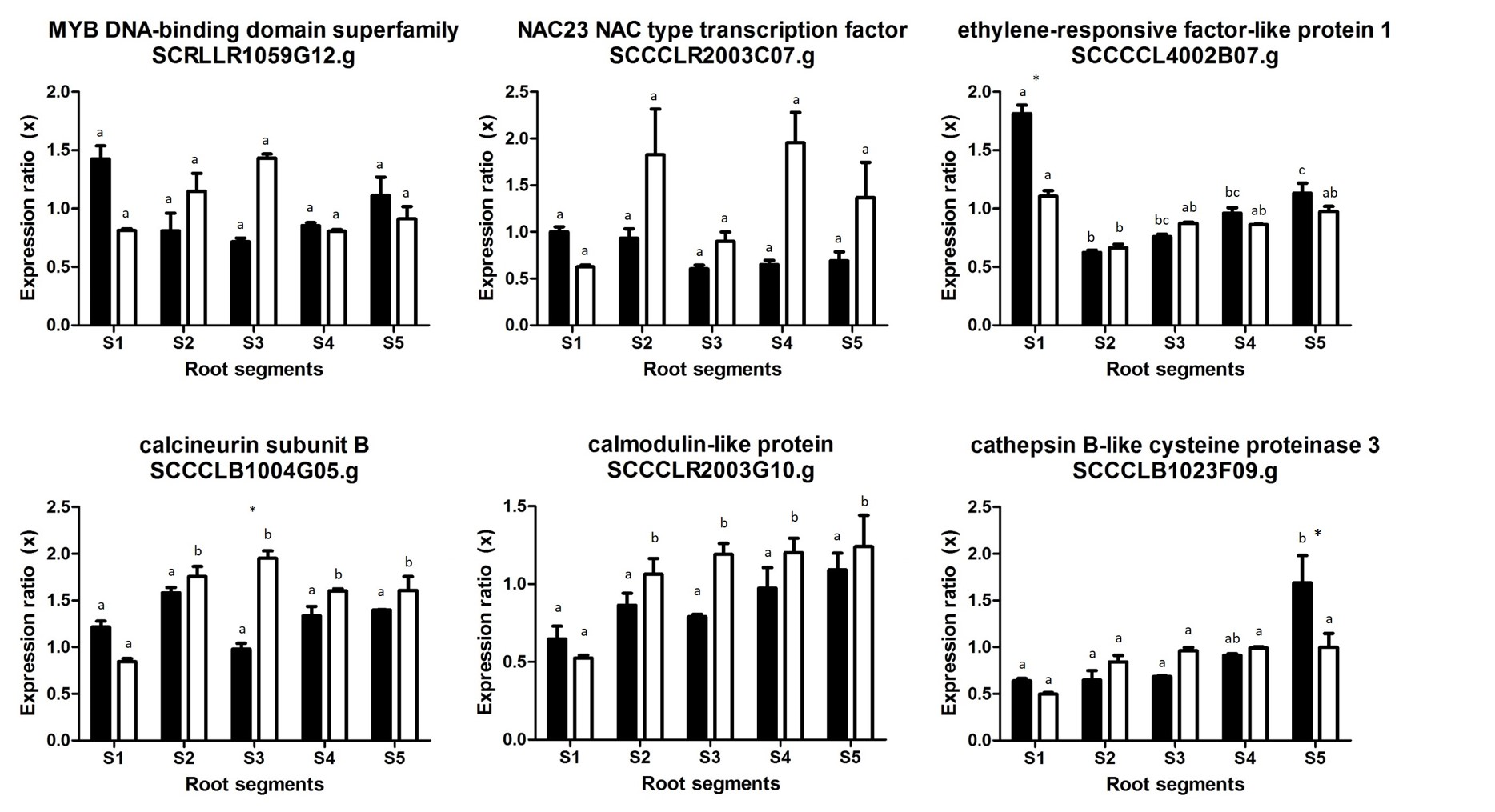

Supplement: Supplementary Figure S1 [file ery362_suppl_supplementary_figure_s1.png]

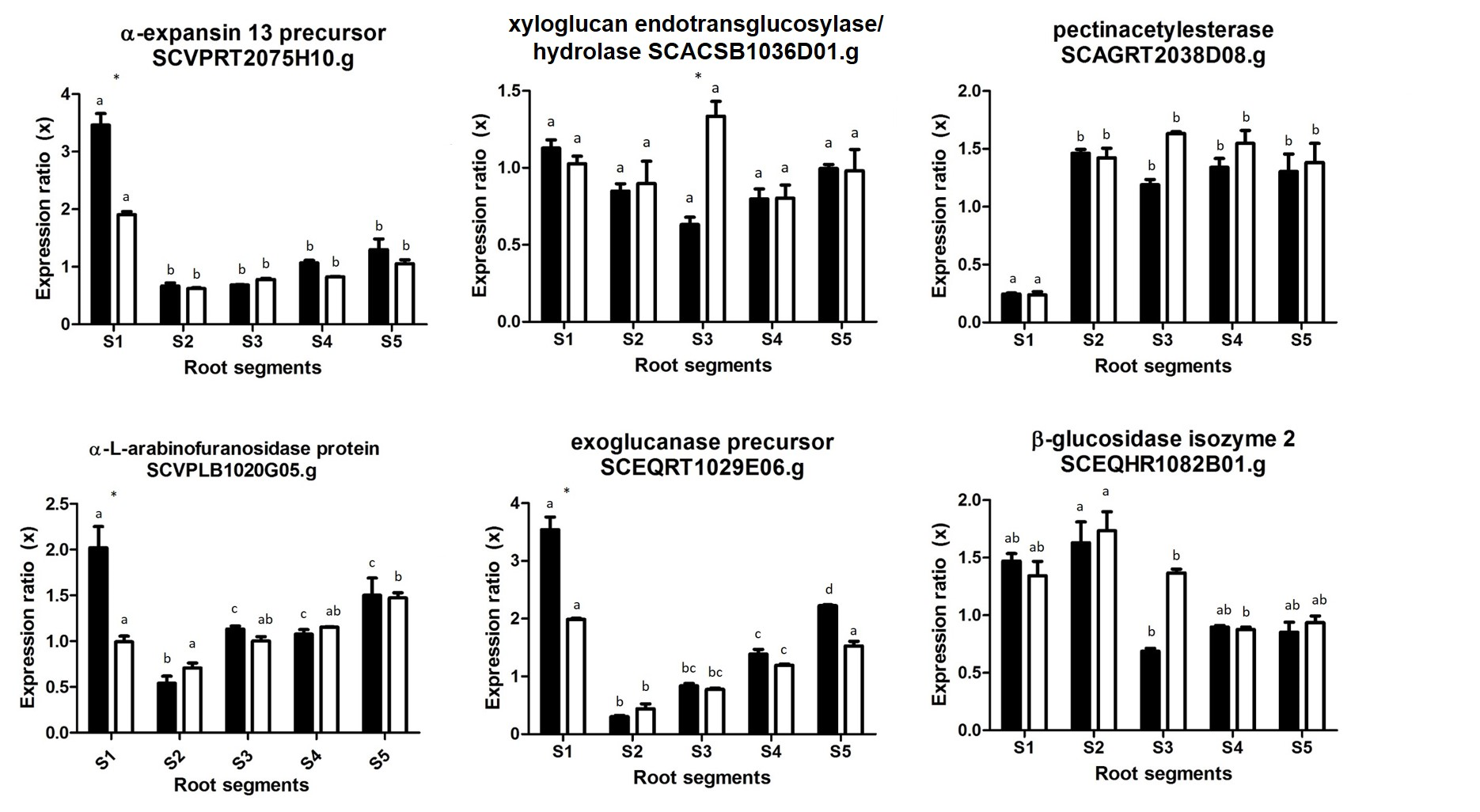

Supplement: Supplementary Figure S2 [file ery362_suppl_supplementary_figure_s2.png]

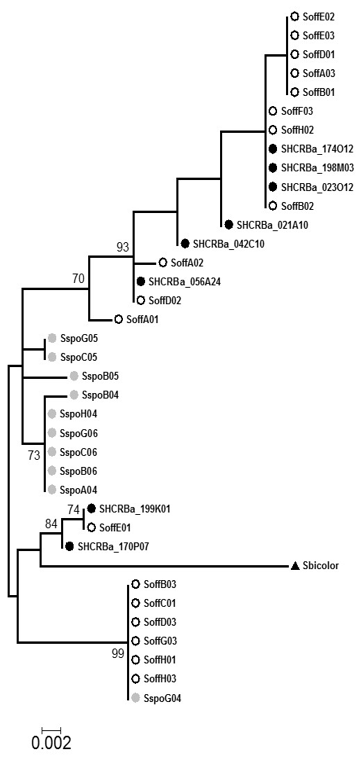

Supplement: Supplementary Figure S3 [file ery362_suppl_supplementary_figure_s3.png]

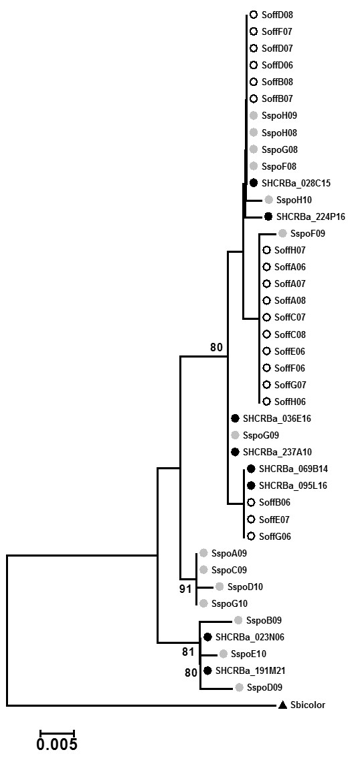

Supplement: Supplementary Figure S4 [file ery362_suppl_supplementary_figure_s4.png]
